# Intracellular imaging of nanoparticles: Is it an elemental mistake to believe what you see?

**DOI:** 10.1186/1743-8977-7-15

**Published:** 2010-06-03

**Authors:** Christina Brandenberger, Martin JD Clift, Dimitri Vanhecke, Christian Mühlfeld, Vicki Stone, Peter Gehr, Barbara Rothen-Rutishauser

**Affiliations:** 1 Institute of Anatomy, University of Bern, Baltzerstrasse 2, CH-3000, Bern 9, Switzerland; 2 Centre for Nano Safety, School of Life Sciences, Edinburgh Napier University, Merchiston Campus, 10 Colinton Road, Edinburgh, EH10 5DT, UK; 3 Max Planck Institute of Biochemistry, Department of Molecular Structural Biology, Martinsried, Germany; 4 Institute of Anatomy and Cell Biology, University of Giessen, Giessen, Germany

## Abstract

In order to understand how nanoparticles (NPs <100 nm) interact with cellular systems, potentially causing adverse effects, it is important to be able to detect and localize them within cells. Due to the small size of NPs, transmission electron microscopy (TEM) is an appropriate technique to use for visualizing NPs inside cells, since light microscopy fails to resolve them at a single particle level. However, the presence of other cellular and non-cellular nano-sized structures in TEM cell samples, which may resemble NPs in size, morphology and electron density, can obstruct the precise intracellular identification of NPs. Therefore, elemental analysis is recommended to confirm the presence of NPs inside the cell. The present study highlights the necessity to perform elemental analysis, specifically energy filtering TEM, to confirm intracellular NP localization using the example of quantum dots (QDs). Recently, QDs have gained increased attention due to their fluorescent characteristics, and possible applications for biomedical imaging have been suggested. Nevertheless, potential adverse effects cannot be excluded and some studies point to a correlation between intracellular particle localization and toxic effects.

J774.A1 murine macrophage-like cells were exposed to NH_2 _polyethylene (PEG) QDs and elemental co-localization analysis of two elements present in the QDs (sulfur and cadmium) was performed on putative intracellular QDs with electron spectroscopic imaging (ESI). Both elements were shown on a single particle level and QDs were confirmed to be located inside intracellular vesicles. Nevertheless, ESI analysis showed that not all nano-sized structures, initially identified as QDs, were confirmed. This observation emphasizes the necessity to perform elemental analysis when investigating intracellular NP localization using TEM.

## Background

The tremendous application potential of nano-sized particles (NPs 1-100 nm; ISO/TS 27687:2008) is in sharp contrast to a growing number of critical reports regarding their potential toxicity. In order to correlate any toxic reaction with a NP type, it is indispensable to investigate if the particles are attached to the cell surface or if they enter cells. If NPs are found in cells, their localization in different compartments such as endosomes, lysosomes, mitochondria, the nucleus or the cytosol, may also provide some answers regarding their potential toxicity.

Transmission electron microscopy (TEM) offers adequate resolution to visualize NPs at a single particle level as well as the ability to determine their localization in different cellular compartments. However, only few particle types, such as gold NPs, show unique characteristics like particle shape and electron density that can be easily recognized within cellular compartments. To confirm the presence of NPs and their localization inside cells, additional elemental analysis of the NP compositions is therefore often required [1]. This can be performed on TEM level by energy filtered TEM, since each chemical element shows a characteristic electron energy loss spectrum.

In this study, elemental analysis was performed on intracellular quantum dots (QDs). Semi-conductor QD nanocrystals [2] have gained increased attention in recent years due to their novel fluorescent characteristics and subsequently, their potential advantages as diagnostic and therapeutic tools [3-5]. Therefore, intensive research has focused upon understanding the potential toxic effects of QDs, prior to their use within such medical applications [3]. This is predominantly due to QDs consisting of a heavy-metal core material, such as cadmium-telluride (CdTe) or cadmium-selenide (CdSe), which is covered by a zinc sulfide (ZnS) shell. Although not fully understood, it is these constituents which have subsequently been suggested as driving QD associated toxicity. The QDs used in this study were coated with NH_2 _polyethylene glycol (PEG) and have previously been shown to cause no cytotoxicity [6] or pro-inflammatory cytokine stimulation in J774.A1 cells after 2 h [7]. However, the NH_2 _PEG QDs do induce an increased intracellular Ca^2+ ^concentration after 30 min and a decreased glutathione level after 2 h exposure with 40 nM QD in this macrophage cell-line [7]. In addition, it has also been shown that the specific intracellular localization (such as within the nucleus, cytosol, mitochondria or vesicles) significantly determines QD toxicity [8,9].

Since QDs are highly fluorescent, research using laser scanning microscopy (LSM) has been used to identify QD intracellular localization via a series of fluorescent markers for intracellular organelles, such as the cytosol, nucleus or intracellular vesicles [9,10]. Despite the advantages of LSM techniques, light microscopic resolution is limited for the size scale of NPs. TEM, however, provides an adequate resolution at a single particle level and, theoretically, due to the heavy-metal core of QDs, TEM is a viable option for determining their intracellular localization. However, the relatively weak electron density of QDs compared to TEM sample staining agents, such as osmium, uranyl acetate and lead citrate, as well as their small size (~5 nm) similar to one of cytoplasmic protein complexes, makes it extremely difficult to detect QDs inside cells. Therefore, electron spectroscopic imaging (ESI) [11] was performed to confirm the intracellular QDs.

## Methods

**Figure 1:**
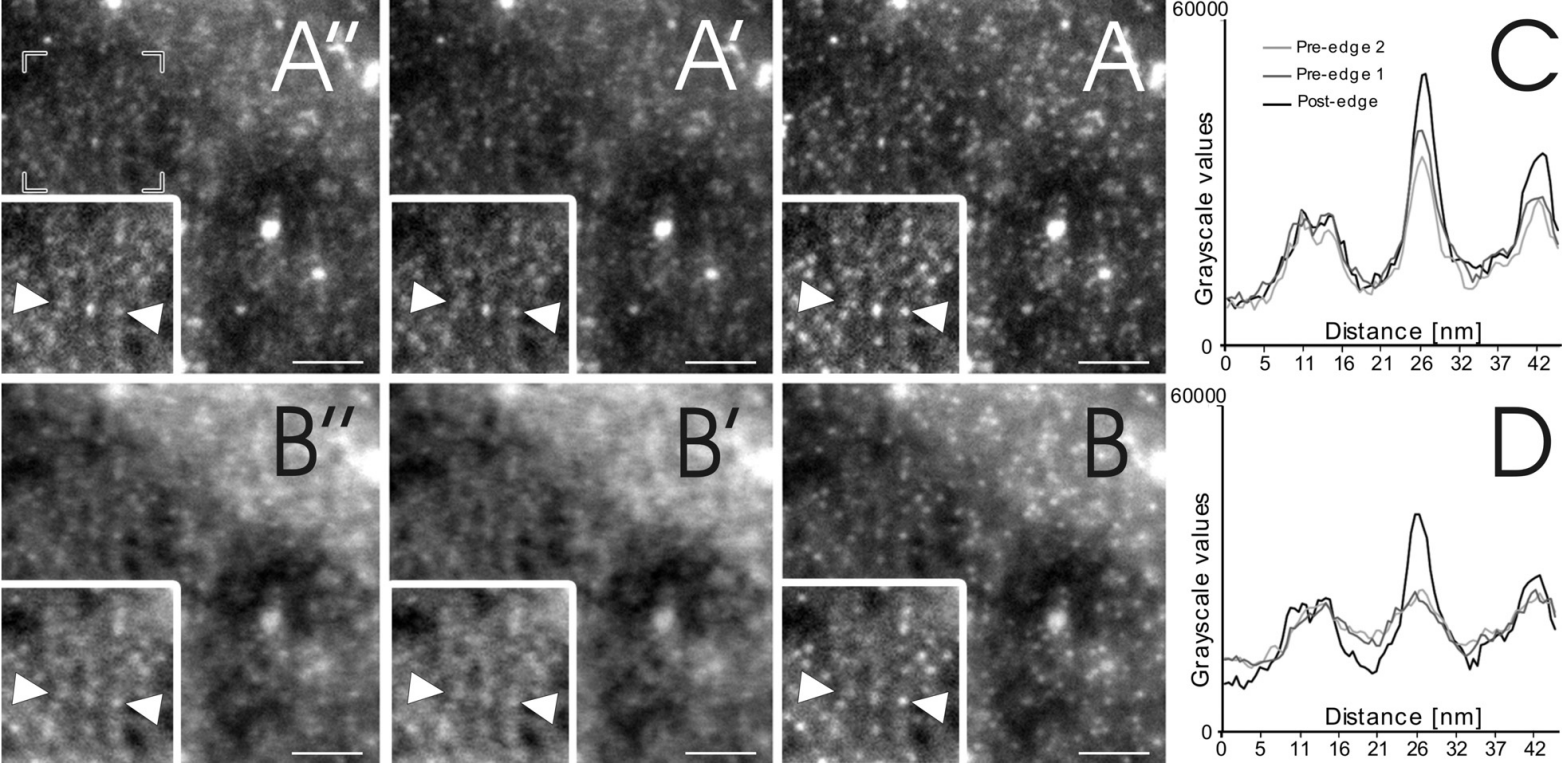
**Electron energy loss micrographs**. Figures A (A-A") and B (B-B") represent the electron energy loss signal of S and Cd respectively. Images A' and B' (pre-edge 1), as well as A" and B" (pre-edge 2), show the background signal of each element, whereas image A and B show the post-edge signal with the highest specific signal peak. In both image series, it is evident that there is a strong primary signal for S and Cd which enables a background subtraction to be performed. To emphasize this fact, figures C (S) and D (Cd) show the grey scale values of the sections between the two arrows, resulting in the strongest signal at the post-edge image: The peak between 20 nm and 30 nm, (black line) indicates that this object is a QD, whereas the peak between 5 nm and 15 nm shows no difference in intensity over the three images, resulting in the conclusion that this object is not a QD. The scale bar equates to 50 nm.

**Figure 2:**
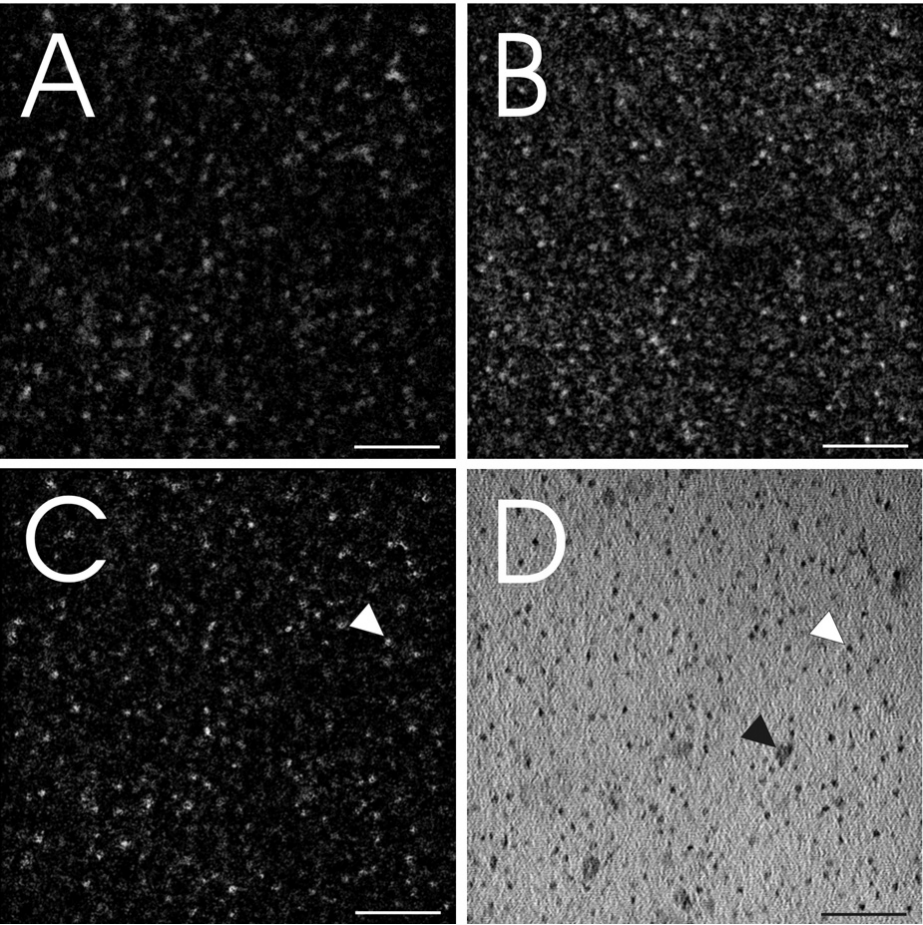
**Electron spectroscopic images of NH_2 _PEG QDs deposited on a TEM grid**. All images are taken at the same area of interest. Images A and B show ESI analysis of S and Cd respectively and figure C represents the signal intersect S/Cd of figure A and B. The bright field image with the QDs is shown in image D. It becomes apparent that not all structures in image D refer to QDs, as there is no corresponding signal in the S/Cd image of figure C (e.g. white arrow = QD, black arrow = non-QD). Scale bars equate to 50 nm.

Imaging and ESI analysis were performed with a Tecnai F20 TEM (FEI, Eindhoven, The Netherlands) equipped with a GIF Tridiem energy filter and Ultrascan 1000 CCD camera (Gatan, Pleasanton, USA). Initially, QDs consisting of a CdTe/CdSe core, covered by a ZnS shell and coated with NH_2 _PEG (Invitrogen, Paisley, UK) were deposited on a TEM grid. Both bright field and ESI images were recorded at a final magnification of 160000× and evaluated using Digital Micrograph Imaging software (Gatan, Pleasanton, USA). ESI images were recorded according to a three-window approach including one post-edge and two pre-edge images [12] (Figure 1). The post-edge represents the signal peak of the electron energy loss of a specific element, whilst the two pre-edge images allow logarithmic regression fitting of the background signal, which is subtracted from the post-edge signal. Sulfur (S) energy loss images were taken at a post-edge of 180eV, pre-edge2 of 145eV, and a pre-edge1 of 155eV. The slit width was 10eV and the exposure time was 6 s, with an image binning of 4 and a cumulative image addition of 6 images per exposure (Figure 1A-A"). Cd energy loss images were taken at a post-edge of 77eV, pre-edge2 of 53eV and pre-edge1 of 61eV. All images were obtained using a slit width of 8eV with a 10 s exposure time, with an image binning of 4 and a cumulative image addition of 8 images per exposure (Figure 1B-B"). For both elements, a stronger signal related to the location area of the QDs can be noted at the post-edge images (Figure 1A and 1B). The graphs in figures 1C and 1D show the grey scale values of the intersection between the two arrows. It becomes apparent that the post-edge image shows the highest intensity at the area of particle localization. Other non-specific signals are not enhanced in the post-edge image compared to the background images. The substitution of the background images from the post-edge image results in the images shown in figures 2A and 2B. Image analysis and reconstruction was performed with ImageJ (open source software; http://rsbweb.nih.gov/ij). For calculating S and Cd image signal intersects as shown in figure 2C, a background reduction (rolling ball radius: 15 pixels) and outliner removing (pixel radius: 1, threshold: 50, bright signal) was performed, followed by an image alignment [13] and an overlap signal extraction. The bright field image of the same position is shown in figure 2D. Note that mass contrast effects of contamination are apparent in figure 2D (black arrowheads) and are present in all three edge windows as well (Figure 1), but not in the resulting ESI windows (Figure 2A-C).

To investigate intracellular particle localization, J774.A1 murine 'macrophage-like' cells were cultured in a 24-well plate, at a density of 2.5 × 10^5 ^cells/mL as previously described [6], and further exposed to 40 nM QDs for 2 h in an environment of 37°C, 5% CO_2_. Investigation of the intracellular localization of the QDs was performed initially via LSM (Zeiss 510 Meta; Axiovert 200 M, Lasers: HeNe 633 nm, and Ar 488 nm), which confirmed that QDs had entered the macrophages [6]. The cells were then fixed with 1 M glutaraldehyde in 0.1 M Na-cacodylate diluted in PBS at pH 7.3, for 3 h at 4°C. The samples were then embedded for TEM by post-fixation in 1% osmium tetroxide in 0.1 M Na-cacodylate buffer for 45 min, washing with 0.1 M Na-cacodylate buffer at 3 and 10 min changes, dehydration in graded concentrations of acetone (50%, 70%, 90% and 100%) and embedded in Araldite resin. The embedded samples were then cut to 60 nm thick ultrathin sections, mounted onto square 400 mesh copper grids (Agar Scientific, Essex, England) and stained with uranyl acetate and lead citrate. The QDs intracellular localization was subsequently investigated using ESI as described before.

## Results and Discussion

**Figure 3:**
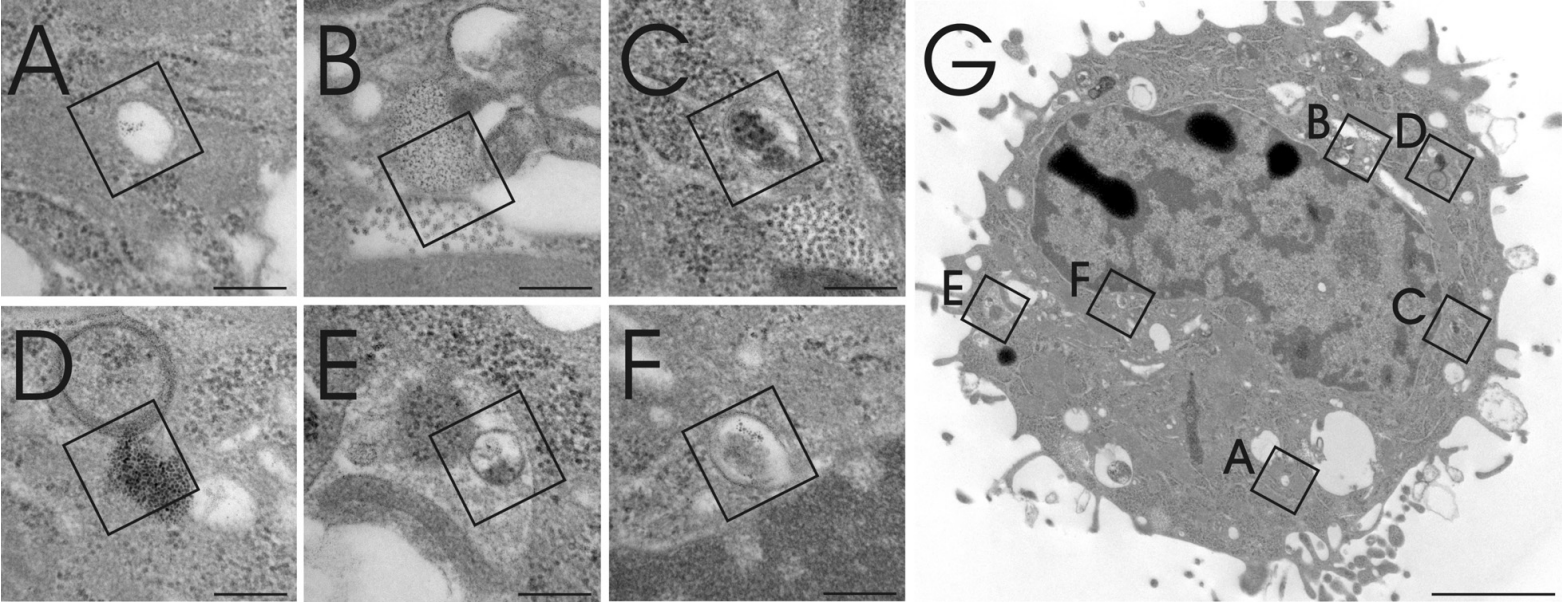
**Image of a J774.A1 murine 'macrophage-like' cell, as observed via TEM**. The macrophage cell was exposed to NH_2 _PEG QDs for 2 h at 40 nM. Six different areas (Figure A-F) possibly containing QDs were recorded by TEM from one selected cell (Figure G). The squares A-F mark the selected area where ESI analysis (assessing the elements S and Cd) was subsequently performed in order to identify and define the presence of QDs. Scale bars A-F equate to 200 nm and G equates to 2 μm.

**Figure 4:**
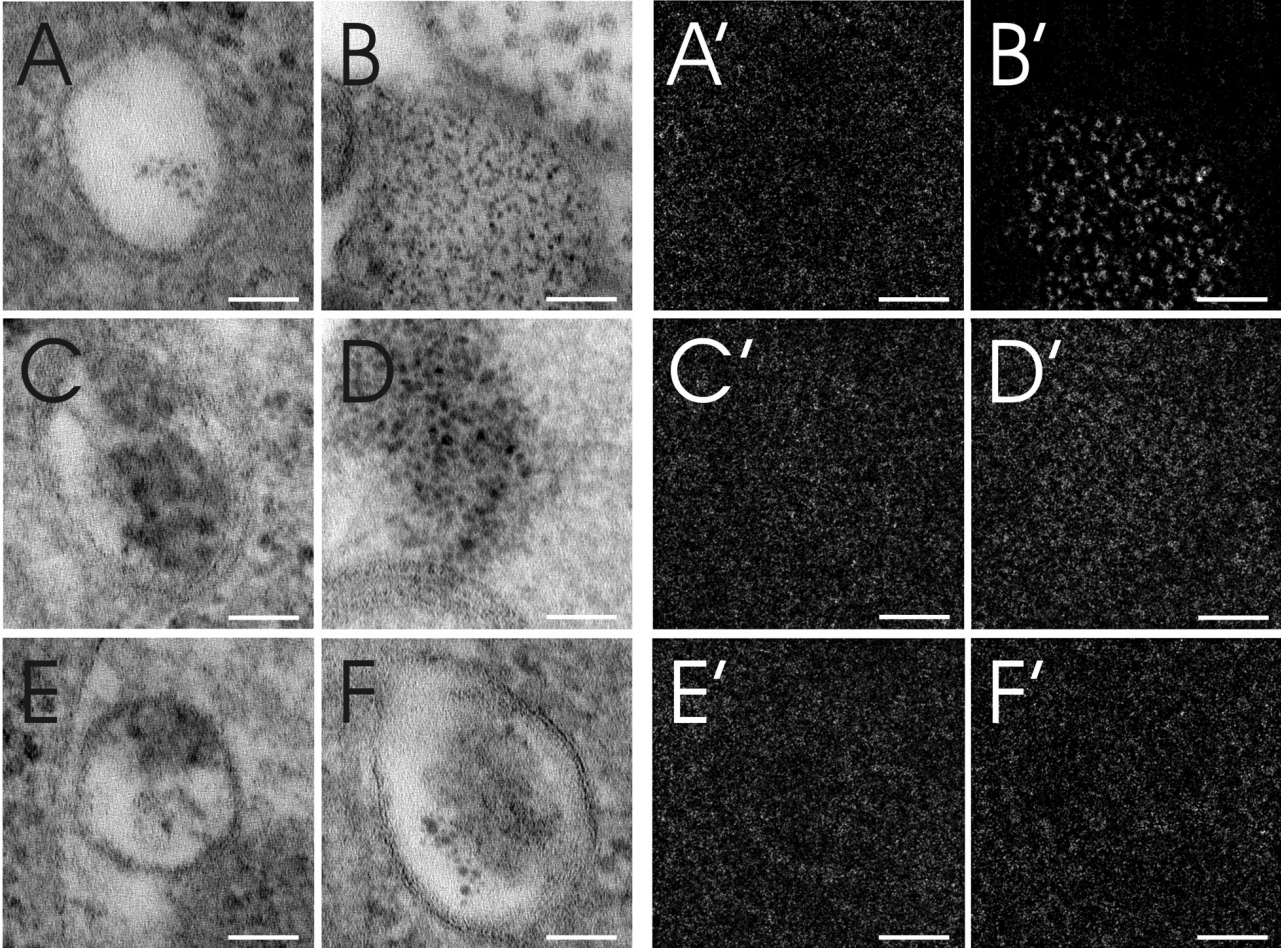
**Different intracellular areas were scanned for S and Cd**. Figures A-F represent the areas selected in figure 3 and figures A'-F' show the corresponding S/Cd ESI image. Image B and B' show a homogenous distribution of QDs within an intracellular vesicle with a specific signal for S/Cd, whereas other images are only related to noisy unspecific S/Cd background. No further analysis on these structural origins was performed, but it is assumed that the structures present in A, D and F are contaminants of TEM embedding and staining with heavy metals, whereas the structures in C and E possibly represent protein or lipid aggregates. All scale bars equate to 50 nm.

Different areas of a macrophage were screened for QDs and six areas, potentially containing QDs, were selected (Figure 3). However, the estimation of the presence of QDs, in any of the selected areas, using visual analysis only was difficult and inconclusive. Following ESI analysis (Figure 4) however, it was identified that, of these six different areas analyzed, only one selected field positively confirmed the presence of QDs (Figure 4B) due to a clear S/Cd signal (Figure 4B'). Analysis of the other five areas only detected background noise (Figures 4A', 4A'-F'). The composition of these structures was not subsequently analyzed and therefore their origin can only be speculated. It is assumed that the structures present in figures 4A, D and 4F are contaminants of the same source from TEM embedding and staining with heavy metals, whereas the structures in figures 4C and 4E may represent protein complexes or other cellular osmiophilic structures.

**Figure 5:**
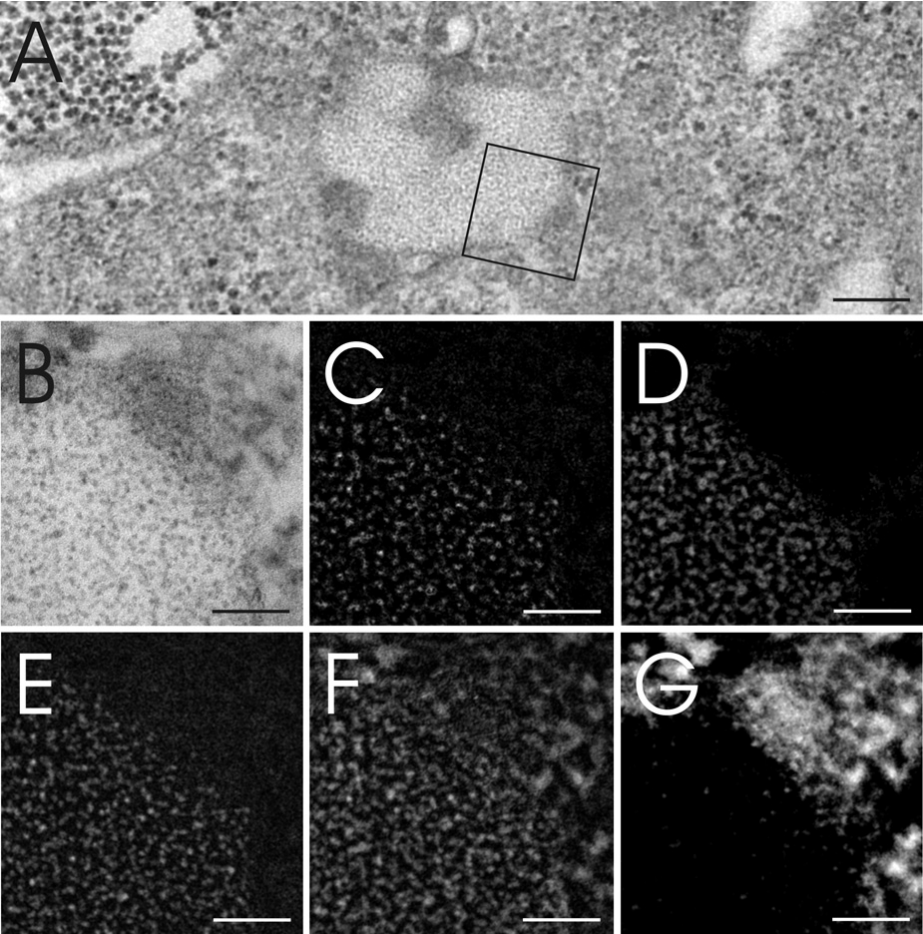
**Elemental analysis of intracellular QDs**. Figure A shows an intracellular vesicle containing QDs and the area analyzed by ESI as shown at higher magnification in figure B. Elemental analysis by ESI for S (E), Cd (F) and Os (G) has been performed. Figure C further represents the ESI signal extraction of S and Se. Figure D represents the Se signal without the Os signal. Scale bar A equates to 200 nm and B-G equate to 50 nm.

Other elements usually contained within QDs, such as Se, Te or Zn, were also investigated. Detection was also possible for Se (post-edge: 67eV, pre-edge2: 51eV, pre-edge1: 43eV, slit width: 8eV, exposure time 6 × 5 s, image binning of 4), resulting in the same position signal as S and Cd. However, within embedded cell samples, some interference with the signal of osmium (Os), a sample staining agent, was observed (Figure 5). This makes Se less suitable for intracellular QD detection. No detection was possible for Te and Zn due to the higher electron energy loss (Te 572eV; Zn 1020eV) than S (165eV), hence the small elemental traces present in the QD samples were too weak to be captured or even confirmed.

**Figure 6:**
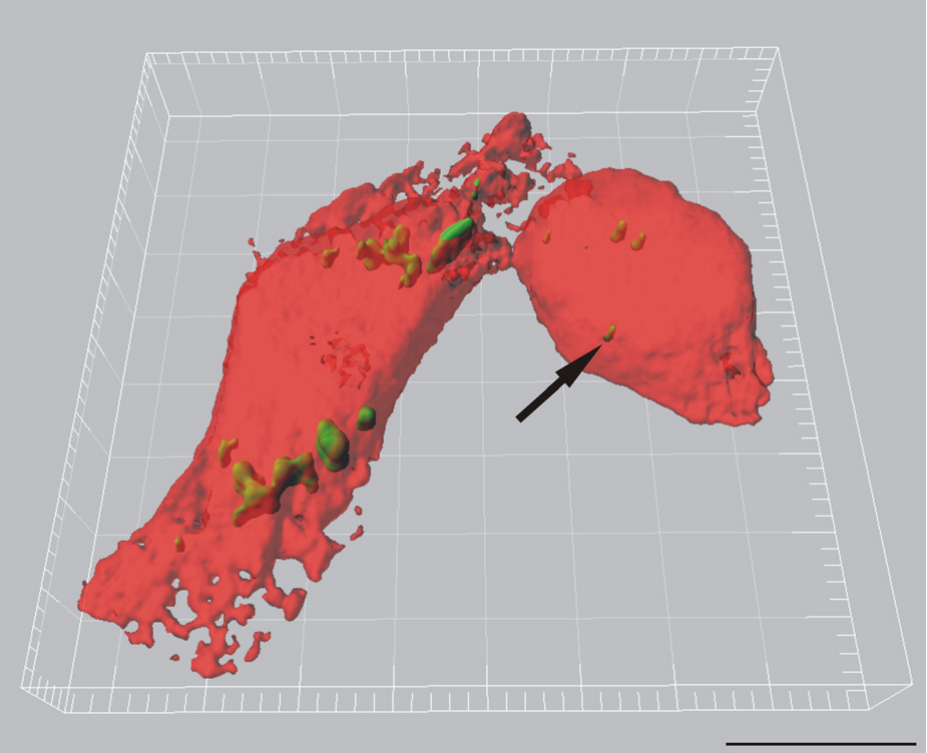
**J774.A1 macrophages (red, transparent volume rendering) containing intracellular NH_2 _PEG QDs (green, surface rendering)**, visualized by LSM and digital image restoration (IMARIS, Bitplane AG, Switzerland). The arrow indicates a small agglomerate of intracellular QDs. However, considering the scales of magnification and the high amount of QDs present per vesicles as shown in figures 4 and 5, it can be concluded that a single detected particle event by LSM usually correspond to a high number of particles detected by TEM. Scale bar equates to 10 μm.

The QDs were shown to be homogeneously distributed inside a cellular vesicle (Figure 4B and 5A). This observation is in accordance with LSM analysis which shows NH_2 _PEG QDs to be located inside endosomes and lysosomes after 2 h (Clift MJD, Brown DM, Brandenberger C, Byrne G, Stolnik-Trenkic S, Rothen-Rutishauser B, and Stone V: The uptake and intracellular fate of a series of different surface coated quantum dots, submitted). It has to be noted however, that the observed QD accumulation at TEM corresponds to a single intracellular signal only at LSM due to limited light microscopic resolution (Figure 6). Hence, quantitative QD analysis by LSM results in a large underestimation of the total intracellular particle number.

The results of this study emphasize the need for better characterization of intracellular NPs, as not all detected electron dense or irregular, nano-sized, intracellular structures represent NPs. Only a limited number of NP types show very unique characteristics, including particle shape and electron density, which can be easily and exclusively recognized within cells. Despite this fact, several studies investigating intracellular localization by TEM have not performed any form of elemental analysis to confirm the presence of intracellular NPs [14-16]. In each example, additional elemental analysis such as ESI or Energy Dispersive X-ray Spectroscopy (EDXS) would be indispensable to the conclusions made by these studies. In light of this fact, statements made within the literature concerning the intracellular localization of NPs without adequate analysis should therefore be taken with caution. Obtaining reliable information pertaining to the intracellular localization of NPs is of increasing importance due to the need to understand NP-cell interactions. As the intracellular localization of NPs has been shown to be related to their toxicity [9], information regarding the precise intracellular localization of NPs is not only imperative in order to understand the potential adverse effects of exposure to NPs, but also to realize the proposed advantages that are posed by nanotechnology.

## Abbreviations

ESI: electron spectroscopic imaging; LSM: laser scanning microscope; NP: nanoparticle; PBS: phosphate buffered saline; PEG: polyethylene glycol; QD: quantum dots; TEM: transmission electron microscope.

## Authors' contributions

CB planned the concept and study design, performed the electron microscopic ESI analysis, interpreted the results and wrote major parts of the manuscript. MJDC planned the concept and study design, performed the cell culture exposure experiments and wrote major parts of the manuscript. DV contributed substantially in establishing the methods, performed parts of ESI image analysis and interpreted the results. CM contributed in planning the concept and design of the study and made substantial contributions to the analysis and interpretation of the data. VS made substantial contributions to the analysis and interpretation of the data. PG made substantial contributions to the analysis and interpretation of the data. BRR planned the concept and study design, made substantial contributions to the analysis and interpretation of the data and wrote parts of the manuscript. All of the authors have critically read the manuscript and approved its submission.

## Competing financial interests

The authors declare that they have no competing interests.
